# DNAJB7 is dispensable for male fertility in mice

**DOI:** 10.1186/s12958-023-01086-6

**Published:** 2023-03-31

**Authors:** Shun Bai, Meihong Hu, Lina Yu, Linjun Chen, Jidong Zhou, Limin Wu, Bo Xu, Xiaohua Jiang, Xindong Zhang, Xianhong Tong, Qiuling Yue

**Affiliations:** 1 Reproductive and Genetic Hospital, The First Affiliated Hospital of USTC, Division of Life Sciences and Medicine, University of Science and Technology of China, Hefei, Anhui 230001 P. R. China; 2 Center for Reproductive Medicine and Obstetrics and Gynecology, Nanjing Drum Tower Hospital, Nanjing University Medical School, Nanjing, Jiangsu 210008 P. R. China

**Keywords:** *Dnajb7*, Spermatogenesis, Male fertility

## Abstract

**Background:**

DNAJBs are highly conserved proteins that are involved in various biological processes. Although several DNAJBs are highly expressed in the testis, the function of DNAJB7 in spermatogenesis and male fertility remains unclear.

**Methods:**

To identify the role of DNAJB7 in the male reproduction process, *Dnajb7-*deficient mice were generated by the CRISPR/Cas9-mediated genome editing system. Histological and immunofluorescence assays were performed to analyze the phenotype of the *Dnajb7* mutants.

**Results:**

DNAJB7 is specifically expressed in haploid germ cells. *Dnajb7* knockout mice are fertile and do not have any detectable defects in Sertoli cells, spermatogonia, meiotic and postmeiotic cells, indicating that DNAJB7 is not essential for spermatogenesis.

**Conclusions:**

Our findings suggest that DNAJB7 is dispensable for male fertility in mice, which could prevent duplicative work by other groups.

**Supplementary Information:**

The online version contains supplementary material available at 10.1186/s12958-023-01086-6.

## Introduction

The production of fertilizing sperm by complete spermatogenesis in the testis is an essential process to maintain male fertility. Normal spermatogenesis is extraordinarily complex with numerous regulatory signals, including self-renewal of spermatogonial stem cells, spermatocyte and spermatid differentiation, and the release of sperm [1]. These step-by-step processes require the dynamic expression of thousands of proteins, of which many are testis-enriching proteins [2]. Accordingly, the control of the posttranscriptional regulation of protein expression, including protein folding and sorting, is critical for the production of high-quality germ cells [3].

Classical DNAJ proteins are involved in protein folding, assembly, translocation, degradation and homeostasis [4]. In mammals, DNAJ proteins contain three domains: the J-domain located at the N-terminus for binding HSP70, the zinc-finger domain consisting of four repeats of CxxCxGxG and the C-terminal domain involved in DNAJ dimerization [5]. All three types of DNAJ proteins are classified by these domains, with DNAJA proteins containing all three domains, whereas DNAJB comprises both the C-terminal domain and J-domain, and DNAJC retains only the latter. Based on the known function of DNAJ proteins as cochaperones to maintain normal proteostasis, the roles of DNAJ proteins in neurodegeneration, tumorigenesis, glucose homeostasis and spermatogenesis have been reported [6, 7]. Among the DNAJ family members, over half of the DNAJB proteins are highly expressed in the testis, signaling their critical role in male fertility [8]. For example, DNAJB13 is an axoneme-associated protein and is required for sperm cilia formation [9, 10]. Both heterozygous and homozygous mutations in DNAJB13 lead to male infertility due to teratozoospermia in humans [11–13]. However, few studies have clarified the function of DNAJB family members in the male reproduction process.

DNAJB7, known as an evolutionarily conserved DNAJB protein, was found to be a novel cancer-testis (CT) antigen due to the failure to detect DNAJB7 protein in normal tissues except for the testis [14]. Previously, a study identified DNAJB7 as a tumor-associated antigen (TAA) and demonstrated its role as a potential immunotherapeutic target [14]. However, the function of the CT protein DNAJB7 in spermatogenesis and male fertility remains unclear and deserves further study. This study aimed to investigate the role of DNAJB7 in the male reproduction process using a *Dnajb7* knockout mouse model generated via CRISPR/Cas9 technology. Although DNAJB7 is a conserved and testis-specifically expressed protein, normal spermatogenesis and male reproduction were found in *Dnajb7*-null mice, which indicates that Dnajb7 is not essential for male fertility in mice.

## Materials and methods

### Animals

Mice were housed in the Animal Center of the Affiliated Drum Tower Hospital of Nanjing University Medical School under controlled air pressure and temperature conditions with free access to food and water. Mouse use and experimental procedures were approved by the Institutional Animal Care and Use Committees of Nanjing Drum Tower Hospital (2021AE01035).

### Generation of *Dnajb7* knockout mice via CRISPR/Cas9 technology

*Dnajb7-*deficient mice in the C57BL/6 genetic background were generated by the CRISPR/Cas9-mediated genome editing system (Cyagen Biosciences, Suzhou, China). The *Dnajb7* exon containing the J-domain was targeted by two sgRNAs (5’-ACTGTTTAAAAGGCCCTCGA-3’ and 5’-TGCCACACTATTTACAAGAA-3’). To obtain knockout mice, Cas9 mRNA and sgRNAs were coinjected into the cytoplasm of fertilized eggs. Genotypes of pups were determined by extracting genomic DNA. After genotyping, the F0 mice underwent serial mating to generate homozygous mutants.

### Genotyping

Tail DNA from offspring was extracted and genotyped using PCR amplification (Tab. S1). The results of Sanger sequencing were analyzed using SnapGene (GSL Biotech, Chicago, IL, USA).

### Fertility test

To investigate fertility in knockout mice, 10-week-old *Dnajb7*^+*/*+^ and *Dnajb7*^*−/−*^ male mice were caged with two 10-week-old *Dnajb7*^+*/*+^ female mice for at least 8 weeks. The average litter size for each mouse line was calculated and recorded.

### Histological analyses

Testes and epididymides from 10-week-old *Dnajb7*^+*/*+^ and *Dnajb7*^*−/−*^ males were removed and fixed in Bouin’s solution overnight. Subsequently, tissues were dehydrated in increasing concentrations of ethanol (70%, 80%, 90%, 100%), cleared with xylene, embedded in paraffin and cut into 5-μm-thick sections, followed by hematoxylin and eosin (H&E; Sigma–Aldrich, USA) staining. For sperm staining, cauda epididymal sperm from 10-week-old male mice were isolated, fixed in 4% PFA, spread on clean glass slides and stained with H&E. Sections were analyzed under a microscope (LEICA DM2500, Germany).

### Sperm counts and motility analyses

For the sperm count test, cauda epididymal sperm from 10-week-old *Dnajb7*^+*/*+^ and *Dnajb7*^*−/−*^ males were released in PBS, fixed in 4% PFA and counted using a hemocytometer. For the sperm motility test, cauda epididymis sperm were resuspended in HTF (human tubal fluid) culture medium and analyzed using a computer-aided sperm analysis (CASA) system (Hamilton Thorne Biosciences, USA).

### Quantitative RT–PCR

Total RNA was extracted from tissues and cells by TRIzol reagent (15596018, Thermo Fisher Scientific, MA, USA). The concentration and purity of RNA were determined by absorbance at 260/280 nm using a NanoDrop 2000 (Thermo Fisher Scientific, MA, USA). RNA was reverse transcribed using 5 × All-In-One RT MasterMix (G492, ABM, Canada). The cDNA was diluted and used for quantitative RT–PCR (qRT–PCR) with SYBR Green Master Mix (Q321, Vazyme, China). *18S* rRNA was used to normalize gene expression. The primer sequences are listed in Table S1.

### Cytoplasmic and nuclear extract preparation

Nuclear and cytoplasmic proteins were extracted from adult testes using a nuclear and cytoplasmic protein extraction kit according to the manufacturer’s instructions (P0027, Beyotime, China). GAPDH and Lamin B1 were used as loading controls for cytoplasmic and nuclear extracts, respectively.

### Western blotting

Tissues were collected from 10-week-old *Dnajb7*^+*/*+^ and *Dnajb7*^*−/−*^ males, lysed in RIPA buffer containing protease inhibitor cocktail for 30 min on ice and centrifuged at 13000 rpm for 15 min at 4 °C. The concentration of proteins was determined by the bicinchoninic acid (BCA) protein assay (E11201, Vazyme, China). A total of 20 µg of protein was loaded and separated on 10% SDS–PAGE gels. The primary antibodies used were as follows: anti-DNAJB7 (diluted 1:1000 in TBST, 18540–1-AP, Proteintech, China), anti-DNAJB13 (diluted 1:1000 in TBST, 25118–1-AP, Proteintech, China), anti-Lamin B1 (diluted 1:10000 in TBST, 12987–1-AP, Proteintech, China), anti-GAPDH (diluted 1:10000 in TBST, 60004–1-Ig, Proteintech, China), anti-β-ACTIN (diluted 1:10000 in TBST, P30002M, Abmart, China), and anti-α-TUBULIN (diluted 1:5000 in TBST, 11224–1-AP, Proteintech, China).

### Cell culture and transfection

The mouse DNAJB7 full-length coding sequence was fused to enhanced green fluorescent protein (EGFP). Human embryonic kidney 293 T cells (HEK293T) were obtained from ATCC (CRL-3216) and cultured in Dulbecco’s modified Eagle medium (DMEM, VivaCell, C3113-0500) supplemented with 10% fetal bovine serum (FBS, Gibco, 16000–044) and 1% penicillin–streptomycin (Gibco, 15140). HEK293T cells were transfected with plasmids harboring EGFP or EGFP-DNAJB7 using a Lipofectamine 3000 transfection kit (Invitrogen, L3000015). Expression was detected 28 h post-transfection under a fluorescence microscope (Nikon ECLIPSE 80i).

### Immunofluorescence

Samples were fixed in 4% paraformaldehyde (PFA), dehydrated in graded ethanol (70-100%) and embedded in paraffin. Sections were blocked in 10% goat serum and incubated with the following primary antibodies: anti-PLZF (diluted 1:100 in TBST, AF-2944, R&D Systems, USA) anti-γH2AX (diluted 1:100 in TBST, 16-202A, Merck Millipore, USA) and anti-SOX9 (diluted 1:100 in TBST, AB5535, Merck Millipore, USA). Nuclear DNA and acrosomes were stained with 4’,6-diamidino-2-phenylindole (DAPI, F6057, Sigma–Aldrich, USA) and FITC-conjugated peanut agglutinin (PNA, RL-1072, Vector Labs, USA), respectively. Sections were analyzed using a fluorescence microscope (Leica DM300, Wetzlar, Germany).

### In-situ hybridization

Testes were embedded in O.C.T compound (Sakura Finetek, Torrance, CA), frozen in liquid nitrogen, and cut into 8-μm-thick sections. The probes (Table S1) were added to the sections and hybridized overnight at 60 °C. After washing and blocking at room temperature, sections were incubated with alkaline phosphatase (AP) conjugated anti-DIG Fab fragments overnight. Sections were cleaned with maleic acid buffer containing Tween 20 (MABT) solution and AP buffer. After adding chromogenic solution (Beyotime Biotechnology, C3206), the sections were washed with double-distilled water, dehydrated in gradient ethanol, cleared with xylene, mounted with SlowFade Gold antifade reagent (Life Technologies), and finally analyzed under a microscope (LEICA DM2500, Germany).

### Transmission electron microscopy

For ultrastructural analysis, samples were fixed in 2.5% glutaraldehyde, postfixed in 1% OsO4, dehydrated in a graded series of ethanol (30%, 50%, 75%, 95%, and 100%), infiltrated with a mixture of acetone and Epon resin, embedded in araldite and sectioned at 60 nm thickness. Ultrathin sections were stained with uranyl acetate and lead citrate. A transmission electron microscope (Tecnai G2; FEI, Eindhoven, The Netherlands) was used to capture images.

### Phylogenetic analyses

Multiple alignments of amino acid sequences were downloaded from the NCBI database, and phylogenetic trees were constructed by MEGA X software using the neighbor-joining method. Multiple alignments were performed using MultAlin (http://multalin.toulouse.inra.fr/multalin/multalin.html).

### Statistical analysis

All data are reported as the mean and standard deviation (SD). Statistical significance was tested by GraphPad Prism 8.0 software using a two-tailed unpaired Student's t test. A *p* value < 0.05 was considered statistically significant.

## Results

### DNAJB7 is expressed specifically in the testis

**Fig. 1:**
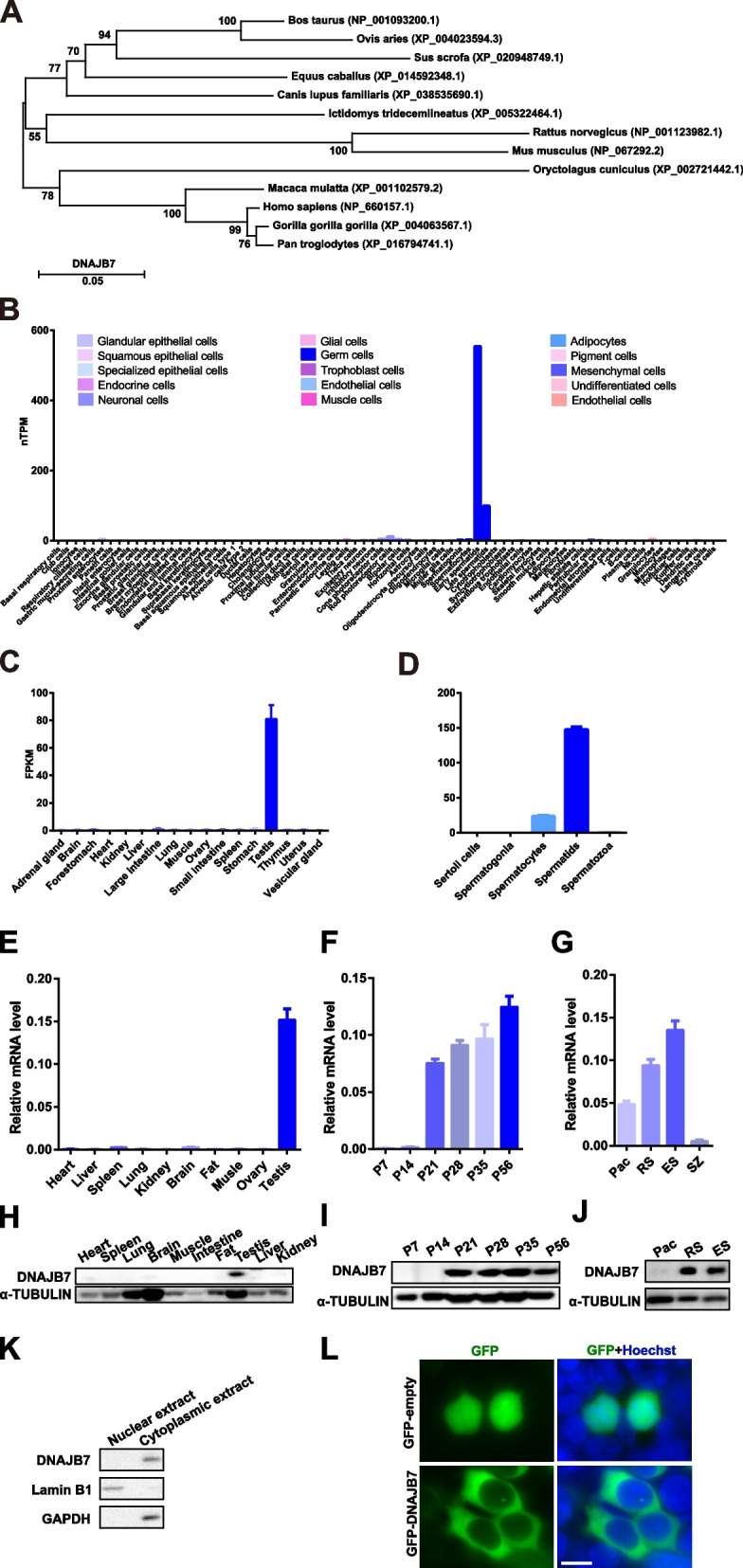
*Dnajb7* is a conserved gene that is highly expressed in postmeiotic germ cells. **A** Phylogenetic trees of DNAJB7 homologous proteins in mammalian species. The numbers in the dendrogram are bootstrap values (%). **B**, **C**, **D** Expression pattern of *Dnajb7* mRNA from published human single-cell RNA-seq data (**B**) and published RNA-seq data of various types of mouse tissues (**C**) and isolated spermatogenetic cells (**D**). **E** qRT–PCR showing the expression pattern of *Dnajb7* mRNA in different tissues of 10-week-old mice, *n* = 3. (F) qRT-PCR analyses of *Dnajb7* mRNA levels in developing testes at postnatal Day 7 (P7), P14, P21, P28, P35 and P56, *n* = 3. **G** qRT-PCR showing the expression pattern of *Dnajb7* mRNA across isolated spermatogenetic cells and mature spermatozoa, *n* = 3. PS, pachytene spermatocytes; RS, round spermatids; ES, elongating spermatids. SZ, spermatozoa. **H** Western blot analyses of the expression pattern of DNAJB7 protein in different tissues of 10-week-old mice. **I** Western blot showing the expression levels of DNAJB7 protein in developing testes at P7, P14, P21, P28, P35 and P56. **J** Western blot analyses of the expression levels of DNAJB7 in spermatogenetic cells isolated at P60. **K** Western blot analyses of DNAJB7 in subcellular fractions of adult testes. Lamin B1 and GAPDH served as nuclear and cytoplasmic protein controls, respectively. **L** Expression of EGFP-DNAJB7 fusion protein in HEK293T cells. Scale bar, 10 μm

Phylogenetic and amino acid alignment analyses identified that DNAJB7 is highly conserved among mammals (Fig. 1A, Fig. S1). To explore the role of DNAJB7 in the reproductive process, we first assessed the tissue- and cell-specific expression patterns of DNAJB7 in humans and mice. Using published single-cell RNA-seq data, *Dnajb7* was found to be enriched in early and late spermatids in humans (Fig. 1B) [15]. Similarly, the mRNA expression of *Dnajb7* was predominantly expressed in the mouse testis, particularly in spermatids among spermatogenic cells (Fig. 1C, D) [8, 16].

We next used quantitative PCR (qPCR) assays to confirm that *Dnajb7* was highly expressed in the mouse testis (Fig. 1E). Additionally, *Dnajb7* was identified to display elevated mRNA expression during spermatogenesis (from postnatal day (P) 7 to P35) (Fig. 1F). Among spermatogenic cells, *Dnajb7* transcripts were predominantly expressed in round spermatids (Fig. 1G), which is in line with a previously published database [16]. At the protein level, DNAJB7 protein was specifically expressed in the testis and was detectable at P21 when early spermatids first appeared and then plateaued (Fig. 1H-J). We further performed nuclear and cytoplasmic protein extraction from adult testes and found that DNAJB7 protein was present in the cytoplasmic extract but undetectable in the nuclear extract (Fig. 1K). Expression of EGFP-DNAJB7 fusion protein in HEK293T cells also indicated that DNAJB7 localized to the cytoplasm (Fig. 1L). Taken together, these results indicate that DNAJB7 is highly enriched in the cytoplasm of spermatids, suggesting a potential role in spermiogenesis and male fertility.

### Generation of *Dnajb7* knockout mice

**Fig. 2:**
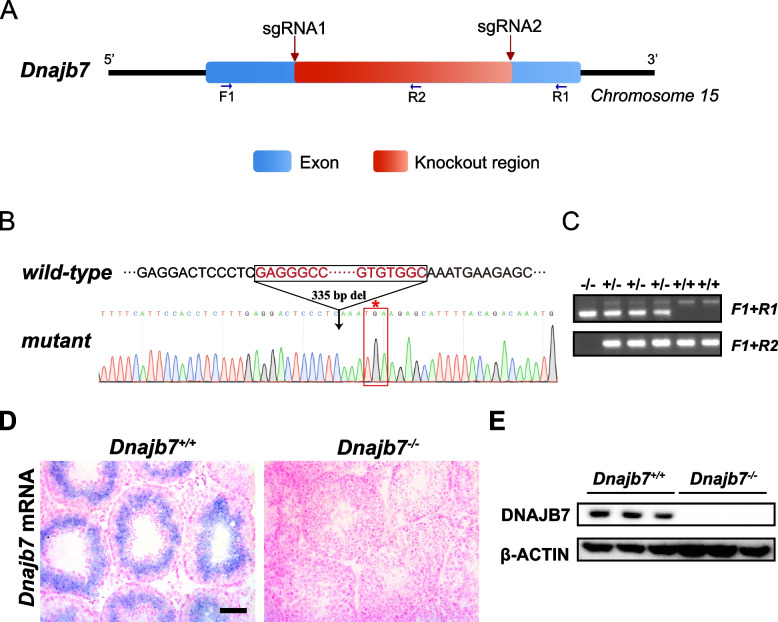
*Dnajb7* knockout mice were generated by CRISPR/Cas9 technology. **A** Schematic diagram of *Dnajb7*^*−/−*^ mouse creation. **B** Sanger sequencing of genomic DNA showing a 335-bp deletion in the *Dnajb7* gene. *, premature stop codon. **C** *Dnajb7*^*−/−*^ mice were identified by genomic PCR. **D** In situ hybridization of *Dnajb7* mRNA in adult *Dnajb7*^+*/*+^ and *Dnajb7*^*−/−*^ testes. *Dnajb7* mRNA was absent in adult *Dnajb7*^*−/−*^ testes but was detectable in adult *Dnajb7*^+*/*+^ testes. Scale bar, 50 μm. **E** DNAJB7 protein was not detected in adult *Dnajb7*^*−/−*^ testes, *n* = 3 for each genotype

To investigate the in vivo function of DNAJB7, we generated *Dnajb7* knockout mice through CRISPR/Cas9 technology and obtained a founder line with a 335-bp deletion, resulting in a frameshift and premature stop codon (Fig. 2A, B). Heterozygous and homozygous mutant *Dnajb7* alleles (hereafter referred to as *Dnajb7*^−/−^) were detected by PCR genotyping of tail-clip genomic DNA (Fig. 2C). In addition, in situ hybridization with a probe against *Dnajb7* mRNA showed absent mRNA in *Dnajb7*^−/−^ testes (Fig. 2D). The absence of DNAJB7 protein in testes from adult *Dnajb7*^−/−^ mice was confirmed by Western blot analyses, indicating that *Dnajb7* knockout mice were successfully generated (Fig. 2E).

### *Dnajb7* mutant males showed normal fertility

**Fig. 3:**
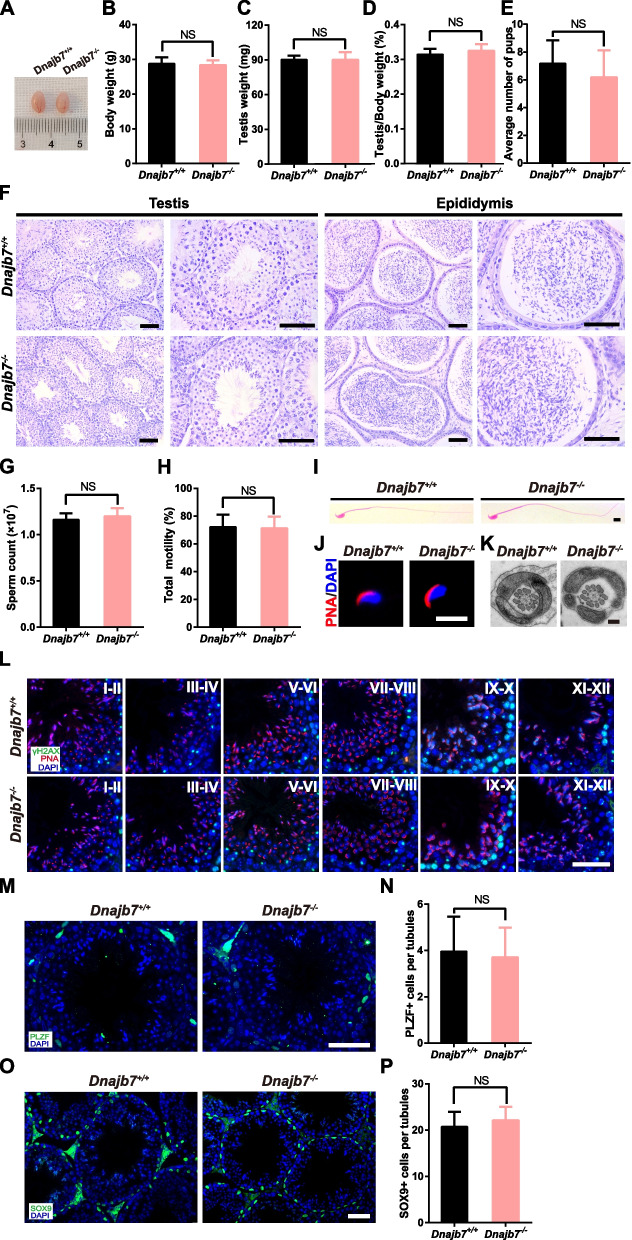
*Dnajb7*^*−/−*^ mice show normal spermatogenesis. **A** Representative image of *Dnajb7*^+*/*+^ and *Dnajb7*^*−/−*^ testes from 10-week-old mice. **B** Body weight of 10-week-old *Dnajb7*^+*/*+^ and *Dnajb7*^*−/−*^ mice. *Dnajb7*^+*/*+^, *n* = 5; *Dnajb7*^*−/−*^, *n* = 4. **C**, **D** Testis weight and the ratio of testis weight to body weight from 10-week-old *Dnajb7*^+*/*+^ and *Dnajb7*^*−/−*^ mice. *Dnajb7*^+*/*+^, *n* = 5; *Dnajb7*^*−/−*^, *n* = 3. **E** Number of pups per litter from *Dnajb7*^+*/*+^ and *Dnajb7*^*−/−*^ males, *n* = 11. **F** H&E staining of testes and epididymides from 10-week-old *Dnajb7*^+*/*+^ and *Dnajb7*^*−/−*^ mice. Scale bar: 50 μm. **G** Sperm count from 10-week-old *Dnajb7*^+*/*+^ and *Dnajb7*^*−/−*^ mice. *Dnajb7*^+*/*+^, *n* = 6; *Dnajb7*^*−/−*^, *n* = 4. **H** Sperm total motility from 10-week-old *Dnajb7*^+*/*+^ and *Dnajb7*^*−/−*^ mice. *Dnajb7*^+*/*+^, *n* = 6; *Dnajb7*^*−/−*^, *n* = 5. **I** H&E staining shows sperm morphology from 10-week-old *Dnajb7*^+*/*+^ and *Dnajb7*^*−/−*^ mice. Scale bar: 10 μm. **J** Immunodetection of PNA in sperm sections from 10-week-old *Dnajb7*^+*/*+^ and *Dnajb7*^*−/−*^ mice. Scale bar: 10 μm. **K** Transmission electronic microscopic analysis of sperm in the cauda epididymis of 10-week-old *Dnajb7*^+*/*+^ and *Dnajb7*^*−/−*^ mice. Scale bar: 200 nm. **L** Immunodetection of γH2AX and PNA in testis sections from 10-week-old *Dnajb7*^+*/*+^ and *Dnajb7*^*−/−*^ mice. Scale bar: 50 μm. **M** Immunostaining of the spermatogonia marker PLZF in testis sections from 10-week-old *Dnajb7*^+*/*+^ and *Dnajb7*^*−/−*^ mice. Scale bar: 50 μm. **N** Quantification of PLZF-positive cells in seminiferous tubules from 10-week-old *Dnajb7*^+*/*+^ and *Dnajb7*^*−/−*^ mice. A total of 30 tubules per genotype were analyzed. **O** Immunostaining of the Sertoli cell marker SOX9 in testis sections from 10-week-old *Dnajb7*^+*/*+^ and *Dnajb7*^*−/−*^ mice. Scale bar: 50 μm. **P** Quantification of SOX9-positive cells in seminiferous tubules from 10-week-old *Dnajb7*^+*/*+^ and *Dnajb7*^*−/−*^ mice. A total of 30 tubules per genotype were analyzed. NS, no significant difference

*Dnajb7*^−/−^ males displayed normal behavior. The body and testicular weights were not different between *Dnajb7*^−/−^ males and wild-type males at 10 weeks of age (Fig. 3A-D). To investigate whether the *Dnajb7* mutant affects male fertility, we performed mating tests and found that *Dnajb7*^−/−^ males were fertile, with litter sizes similar to those of *Dnajb7*^+/+^ males (Fig. 3E). In addition, all spermatogenic cells in seminiferous tubules and mature spermatozoa were observed in *Dnajb7*^−/−^ mice by H&E staining (Fig. 3F). We further detected the long-term consequence of *Dnajb7* deficiency on male fertility. Similarly, normal spermatogenesis and male fertility were found in *Dnajb7*^−/−^ males at 8 months of age (Fig. S3). There were no significant differences in the counts and motility of epididymal sperm between 10-week-old *Dnajb7*^*−/−*^ mice and *Dnajb7*^+/+^ controls (Fig. 3G, H). HE staining also showed that sperm from *Dnajb7*^*−/−*^ males exhibited normal morphology (Fig. 3I). Immunodetection of PNA in sperm sections indicated that *Dnajb7*^*−/−*^ sperm displayed normal acrosome (Fig. 3J). Ultrastructurally, *Dnajb7*^*−/−*^ sperm flagella showed a typical “9 + 2” arrangement of microtubule doublets (Fig. 3K). To study whether germ cell development is affected in *Dnajb7*^−/−^ mice, we performed immunostaining for γ-H2AX-positive spermatocytes and PNA-positive acrosomes in spermatids (Fig. 3L). All stages of spermatogenic cells were observed in testis sections from 10-week-old wild-type mice and *Dnajb7*^−/−^ male mice. We also found that the number of PLZF-positive cells (spermatogonia marker) and SOX9-positive cells (Sertoli cell marker) was similar between wild-type and *Dnajb7*^*−/−*^ seminiferous tubules (Fig. 3M-P).

### Functional redundancy of DNAJBs

**Fig. 4:**
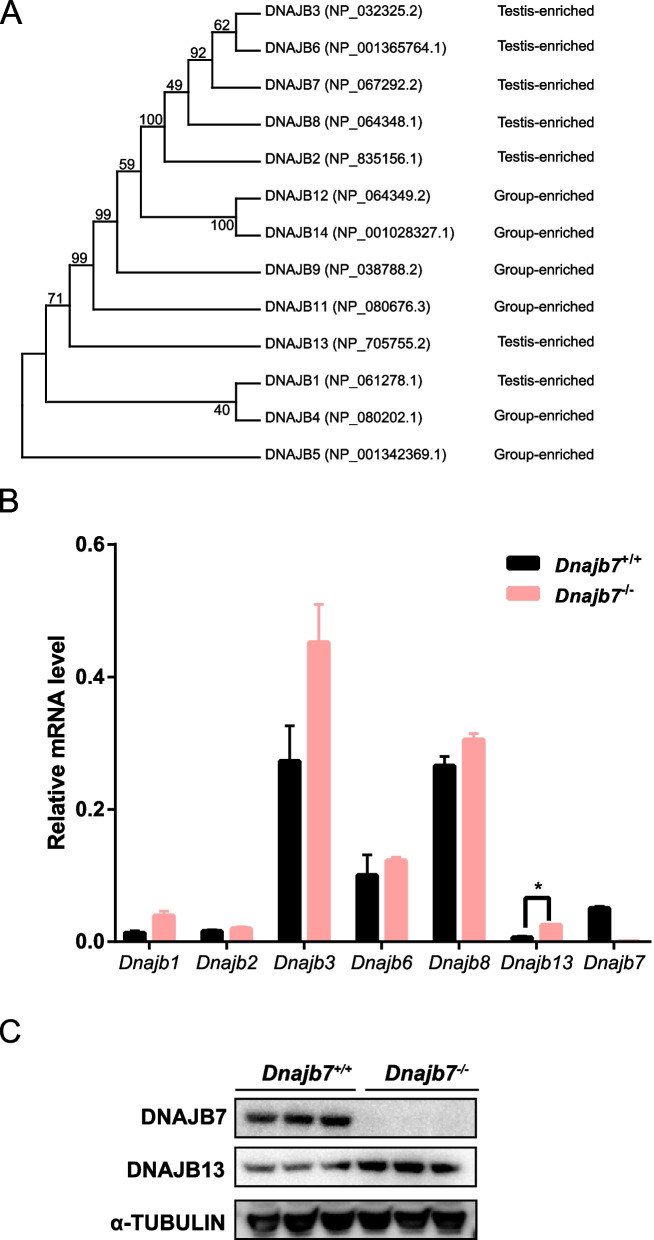
Functional compensation of DNAJBs. **A** Phylogenetic clustering by MEGA X software. **B** QRT-PCR showing the mRNA expression levels of 6 *Dnajb* genes in testes from 10-week-old *Dnajb7*^+*/*+^ and *Dnajb7*^*−/−*^ mice. *P < 0.05. **C** Western blotting showing the level of DNAJB13 protein expression in testes from 10-week-old *Dnajb7*^+*/*+^ and *Dnajb7*^*−/−*^ mice

To further investigate why DNAJB7 is not essential to male fertility, we conducted a phylogenetic analysis of all DNAJB proteins. DNAJB7 was clustered from testis-enriched expressed DNAJB3 and DNAJB6 with a high bootstrap value of 92% (Fig. 4A). Additionally, the expression of *Dnajb1*, *Dnajb2*, *Dnajb3*, *Dnajb6*, *Dnajb8*, and *Dnajb13* were slightly upregulated in the testis of *Dnajb7*^−/−^ mice compared with that in the testis of wild-type mice, although there was no significant difference except for *Dnajb13* (Fig. 4B). Upregulation of DNAJB13 expression was further confirmed by Western blotting (Fig. 4C). In contrast, the transcription level of *Dnajb7* was markedly reduced in the testis of *Dnajb7* mice (Fig. 4B). Together, our findings suggest that DNAJB7 function in male fertility could be compensated by other *Dnajb* family members.

## Discussion

Here, we investigated the function of the cancer-testis gene *Dnajb7* in spermatogenesis and male fertility. Although DNAJB7 was expressed specifically in the testis, we did not find an abnormal reproduction process in *Dnajb7* knockout males. Using histological and immunofluorescence assays, we found all stages of spermatogenic cells in testicular tubules and mature sperm with normal counts and morphology in epididymal tubules in *Dnajb7* mutants.

A number of DNAJ proteins have been identified and characterized in male reproduction [3]. Among the DNAJA proteins, DNAJA4 is predominantly expressed in the mouse testis and heart [17]. Upregulation of *Dnaja4* transcripts was found after heat stress in the rooster testis, indicating its role in protecting spermatogenesis from heat stress [18]. In addition to the DNAJA family, several DNAJC variants were identified as testis-enriched expressed proteins. For example, DNAJC2, a critical epigenetic factor, was detected only in the testis among normal human tissues [19, 20]. Decreased *Dnajc4* mRNA expression levels were found in spermatozoa from infertile men compared with those from fertile men [21]. Additionally, the small mitochondrial protein DNAJC15 was highly detected in the mouse testis [22], while the ribosomal protein DNAJC21 was mainly expressed in meiotic germ cells [8, 23]. It is worth noting that DNAJBs are divided into a group with 13 homologs in mice, of which over half have functions in male reproduction. For example, DNAJB1 protein was mainly located in the sperm head and tail [24]. *Dnajb2* and *Dnajb6* transcripts were mainly expressed in meiotic germ cells, while high levels of *Dnajb3*, *Dnajb8* and *Dnajb13* mRNA were found in postmeiotic germ cells [8]. More importantly, mutations in DNAJB13 cause severe oligo-astheno-teratozoospermia and male infertility in humans [13]. However, in this study, normal fertility was found in *Dnajb7-*deficient males. In addition, there were no defects in spermatogonic cells (PLZF-positive spermatogonia, γH2AX-positive spermatocytes and PNA-positive spermatids) or SOX9-positive Sertoli cells between *Dnajb7*^−/−^ and *Dnajb7*^+/+^ mice. Given that many DNAJs are testis-enriched proteins, the redundant function of DNAJs may result in normal fertility in *Dnajb7*-null males. Similar to DNAJB7, *Dnajb8* is predominantly in haploid male germ cells but dispensable for spermatogenesis [25], although *Dnajb8* transcripts are downregulated in the spermatozoa of infertile men compared to those of fertile men [26]. Functional redundancy of DNAJs has been reported in yeast [27], indicating that DNAJ proteins work in parallel to ensure normal reproduction processes in males.

It should be noted that we found that *Dnajb7* is not required for male fertility only under normal laboratory mating conditions, indicating that subtle phenotypes could not be found in *Dnajb7-*null mice. Some stress conditions, such as heat stress, may induce spermatogenesis defects in *Dnajb7* knockout mice, and these possibilities need further investigation.

In summary, we generated *Dnajb7* knockout mice and demonstrated that mouse DNAJB7 is not essential for spermatogenesis and male fertility, although *Dnajb7* is a highly conserved and testis-specifically expressed gene. We reported this phenotypic information, which could provide basic information for further studies.

## Supplementary Information

**Additional file 1:** **Supplementary figure 1.** Alignment of DNAJB7 protein sequences among mammals. Highly conserved regions are shown in red. Unconserved residues are shown in blue or as asterisks in the consensus. Multiple alignments were performed using MultAlin (http://multalin.toulouse.inra.fr/multalin/multalin.html). **Supplementary figure 2.** The relative amounts of proteins were determined by SDS-PAGE combined with Coomassie blue staining. **Supplementary figure 3.** *Dnajb7*^*−/−*^ males at 8 months of age displayed normal male fertility. (A) Representative image of *Dnajb7*^*+/+*^ and *Dnajb7*^*-/-*^ testes from 8-month-old mice. (B) Number of pups per litter from *Dnajb7*^*+/+*^ and *Dnajb7*^*−/−*^ males at 8 months of age, *n*=11. (C) H&E staining of testes and epididymides from 8-month-old *Dnajb7*^*+/+*^ and *Dnajb7*^*−/−*^ mice. Scale bar: 50 μm. **Supplementary table 1.** List of primer sequences.

## Data Availability

The datasets analyzed during this study are available from the corresponding author.

## References

[1] Nishimura H, L'Hernault SW (2017). Spermatogenesis. Curr Biol.

[2] Uhlen M, Fagerberg L, Hallstrom BM, Lindskog C, Oksvold P, Mardinoglu A, Sivertsson A, Kampf C, Sjostedt E, Asplund A (2015). Proteomics. Tissue-based map of the human proteome. Science.

[3] Meccariello R, Chianese R, Ciaramella V, Fasano S, Pierantoni R (2014). Molecular chaperones, cochaperones, and ubiquitination/deubiquitination system: involvement in the production of high quality spermatozoa. Biomed Res Int.

[4] Zarouchlioti C, Parfitt DA, Li W, Gittings LM, Cheetham ME. DNAJ Proteins in neurodegeneration: essential and protective factors. Philos Trans R Soc Lond B Biol Sci. 2018;373:20160534. 10.1098/rstb.2016.0534 PMC5717533 29203718

[5] Cheetham ME, Caplan AJ (1998). Structure, function and evolution of DnaJ: conservation and adaptation of chaperone function. Cell Stress Chaperones.

[6] Kusumoto H, Hirohashi Y, Nishizawa S, Yamashita M, Yasuda K, Murai A, Takaya A, Mori T, Kubo T, Nakatsugawa M (2018). Cellular stress induces cancer stem-like cells through expression of DNAJB8 by activation of heat shock factor 1. Cancer Sci.

[7] Diane A, Abunada H, Khattab N, Moin ASM, Butler AE, Dehbi M (2021). Role of the DNAJ/HSP40 family in the pathogenesis of insulin resistance and type 2 diabetes. Ageing Res Rev.

[8] Li B, Qing T, Zhu J, Wen Z, Yu Y, Fukumura R, Zheng Y, Gondo Y, Shi L (2017). A comprehensive mouse transcriptomic BodyMap across 17 Tissues by RNA-seq. Sci Rep.

[9] Oji A, Noda T, Fujihara Y, Miyata H, Kim YJ, Muto M, Nozawa K, Matsumura T, Isotani A, Ikawa M (2016). CRISPR/Cas9 mediated genome editing in ES cells and its application for chimeric analysis in mice. Sci Rep.

[10] Guan J, Yuan L (2008). A heat-shock protein 40, DNAJB13, is an axoneme-associated component in mouse spermatozoa. Mol Reprod Dev.

[11] Liu M, Li J, Jiang C, Zhou Y, Sun Y, Yang Y, Shen Y (2022). A novel homozygous mutation in DNAJB13-a gene associated with the sperm axoneme-leads to teratozoospermia. J Assist Reprod Genet.

[12] Li WN, Zhu L, Jia MM, Yin SL, Lu GX, Liu G (2020). Missense mutation in DNAJB13 gene correlated with male fertility in asthenozoospermia. Andrology.

[13] El Khouri E, Thomas L, Jeanson L, Bequignon E, Vallette B, Duquesnoy P, Montantin G, Copin B, Dastot-Le Moal F, Blanchon S (2016). Mutations in DNAJB13, encoding an HSP40 family member, cause primary ciliary dyskinesia and male infertility. Am J Hum Genet.

[14] Scurr MJ, Greenshields-Watson A, Campbell E, Somerville MS, Chen Y, Hulin-Curtis SL, Burnell SEA, Davies JA, Davies MM, Hargest R (2020). Cancer antigen discovery is enabled by RNA sequencing of highly purified malignant and nonmalignant cells. Clin Cancer Res.

[15] Karlsson M, Zhang C, Mear L, Zhong W, Digre A, Katona B, Sjostedt E, Butler L, Odeberg J, Dusart P, et al. A single-cell type transcriptomics map of human tissues. Sci Adv. 2021;7:eabh2169. 10.1126/sciadv.abh2169 PMC8318366 34321199

[16] Soumillon M, Necsulea A, Weier M, Brawand D, Zhang X, Gu H, Barthes P, Kokkinaki M, Nef S, Gnirke A (2013). Cellular source and mechanisms of high transcriptome complexity in the mammalian testis. Cell Rep.

[17] Abdul KM, Terada K, Gotoh T, Hafizur RM, Mori M (2002). Characterization and functional analysis of a heart-enriched DnaJ/ Hsp40 homolog dj4/DjA4. Cell Stress Chaperones.

[18] Wang SH, Cheng CY, Tang PC, Chen CF, Chen HH, Lee YP, Huang SY (2013). Differential gene expressions in testes of L2 strain Taiwan country chicken in response to acute heat stress. Theriogenology.

[19] Aloia L, Demajo S, Di Croce L (2015). ZRF1: a novel epigenetic regulator of stem cell identity and cancer. Cell Cycle.

[20] Greiner J, Ringhoffer M, Taniguchi M, Hauser T, Schmitt A, Dohner H, Schmitt M (2003). Characterization of several leukemia-associated antigens inducing humoral immune responses in acute and chronic myeloid leukemia. Int J Cancer.

[21] Garrido N, Martinez-Conejero JA, Jauregui J, Horcajadas JA, Simon C, Remohi J, Meseguer M (2009). Microarray analysis in sperm from fertile and infertile men without basic sperm analysis abnormalities reveals a significantly different transcriptome. Fertil Steril.

[22] Mitra A, Shevde LA, Samant RS (2009). Multi-faceted role of HSP40 in cancer. Clin Exp Metastasis.

[23] Tummala H, Walne AJ, Williams M, Bockett N, Collopy L, Cardoso S, Ellison A, Wynn R, Leblanc T, Fitzgibbon J (2016). DNAJC21 mutations link a cancer-prone bone marrow failure syndrome to corruption in 60S ribosome subunit maturation. Am J Hum Genet.

[24] Doiguchi M, Kaneko T, Urasoko A, Nishitani H, Iida H (2007). Identification of a heat-shock protein Hsp40, DjB1, as an acrosome- and a tail-associated component in rodent spermatozoa. Mol Reprod Dev.

[25] Wang F, Kong S, Hu X, Li X, Xu B, Yue Q, Fu K, Ye L, Bai S (2020). Dnajb8, a target gene of SOX30, is dispensable for male fertility in mice. PeerJ.

[26] Montjean D, De La Grange P, Gentien D, Rapinat A, Belloc S, Cohen-Bacrie P, Menezo Y, Benkhalifa M (2012). Sperm transcriptome profiling in oligozoospermia. J Assist Reprod Genet.

[27] Sahi C, Craig EA (2007). Network of general and specialty J protein chaperones of the yeast cytosol. Proc Natl Acad Sci U S A.

