# Supplementary material for: DNAJB7 is dispensable for male fertility in mice
Source: Reprod Biol Endocrinol. 2023 Mar 31;21:32. doi: 10.1186/s12958-023-01086-6 (PMC10064739; doi:10.1186/s12958-023-01086-6)
Supplement: Supplementary file 1 — Additional file 1: Supplementary figure 1. Alignment of DNAJB7 protein sequences among mammals. Highly conserved regions are shown in red. Unconserved residues are shown in blue or as asterisks in the consensus. Multiple alignments were performed using MultAlin (http://multalin.toulouse.inra.fr/multalin/multalin.html). Supplementary figure 2. The relative amounts of proteins were determined by SDS-PAGE combined with Coomassie blue staining. Supplementary figure 3. Dnajb7−/− males at 8 months of age displayed normal male fertility. (A) Representative image of Dnajb7+/+ and Dnajb7-/- testes from 8-month-old mice. (B) Number of pups per litter from Dnajb7+/+ and Dnajb7−/− males at 8 months of age, n=11. (C) H&E staining of testes and epididymides from 8-month-old Dnajb7+/+ and Dnajb7−/− mice. Scale bar: 50 μm. Supplementary table 1. List of primer sequences. [file 12958_2023_1086_MOESM1_ESM.zip › Figure S2.pdf]

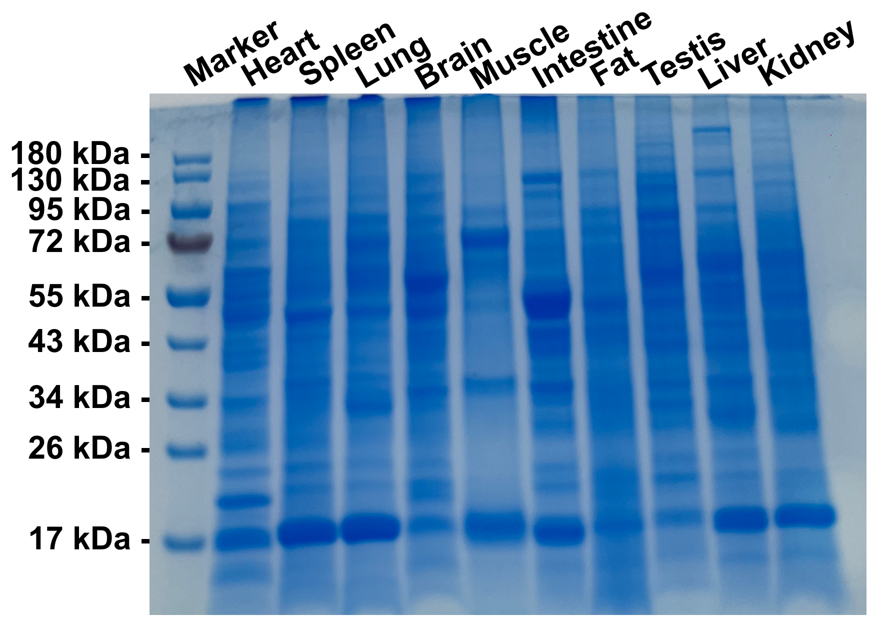
Marker
Heart
Spleen
Lung
Brain
Muscle
Intestine
Fat
Testis
Liver
Kidney
180 kDa
130 kDa
95 kDa
72 kDa
55 kDa
43 kDa
34 kDa
26 kDa
17 kDa
